# Obtaining Polyacrylonitrile Carbon Nanofibers by Electrospinning for Their Application as Flame-Retardant Materials

**DOI:** 10.3390/polym17091255

**Published:** 2025-05-05

**Authors:** Elizaveta Mokhova, Mariia Gordienko, Natalia Menshutina, Ksenia Serkina, Igor Avetissov

**Affiliations:** 1 The Department of Chemical and Pharmaceutical Engineering, Mendeleev University of Chemical Technology of Russia (MUCTR), Moscow 125047, Russia; mokhova.e.k@muctr.ru (E.M.); gordienko.m.g@muctr.ru (M.G.); 2 The Department of Chemistry and Technology of Crystals, Mendeleev University of Chemical Technology of Russia (MUCTR), Moscow 125047, Russia; serkina.k.s@muctr.ru (K.S.); avetisov.i.k@muctr.ru (I.A.)

**Keywords:** electrospinning, micro- and nanofibers, carbon nanofibers, polyacrylonitrile, flame-retardant fibers, titanium dioxide

## Abstract

The article describes obtaining polyacrylonitrile (PAN) nanofibers by electrospinning on a setup developed at the Mendeleev University of Chemical Technology of Russia (MUCTR). A technique for producing PAN-based carbon nanofibers (CNFs) and PAN-based CNFs modified with titanium oxide (TiO_2_) is presented. The article presents a comprehensive study of the characteristics of PAN-based nanofibers and CNFs, including an analysis of the external structure of the fibers, the dependence of fiber diameters on the viscosity of the initial solutions, the effect of temperature treatment on the functional groups of PAN, elemental analysis, and flame-retardant properties. It was found that the fiber diameter and its external structure strongly depend on the viscosity of the initial solutions; an increase in viscosity leads to a linear increase in the fiber diameter. Preliminary temperature treatment at 250 °C helps stabilize PAN nanofibers and prevents their melting at the carbonization stage. The differential scanning calorimetry results allowed us to determine the presence of peaks for the initial PAN nanofibers, indicating an exothermic process in the temperature range of 290–320 °C. The peak height decreased with increasing TiO_2_ concentration in the samples. For CNF samples of different compositions, the endothermic effect prevailed in the temperature range of 400–700 °C, indicating the possible flame-retardant properties of these materials. The limiting oxygen index (LOI) was calculated based on the thermogravimetric analysis results. The highest LOI values were obtained for CNFs based on PAN without adding TiO_2_ nanoparticles and CNFs modified with TiO_2_ (3 wt.%). The resulting CNF-based nonwovens can be recommended for use in heat-protective clothing, flame-retardant mattresses, and flame-retardant suits for the military.

## 1. Introduction

**Figure 1:**
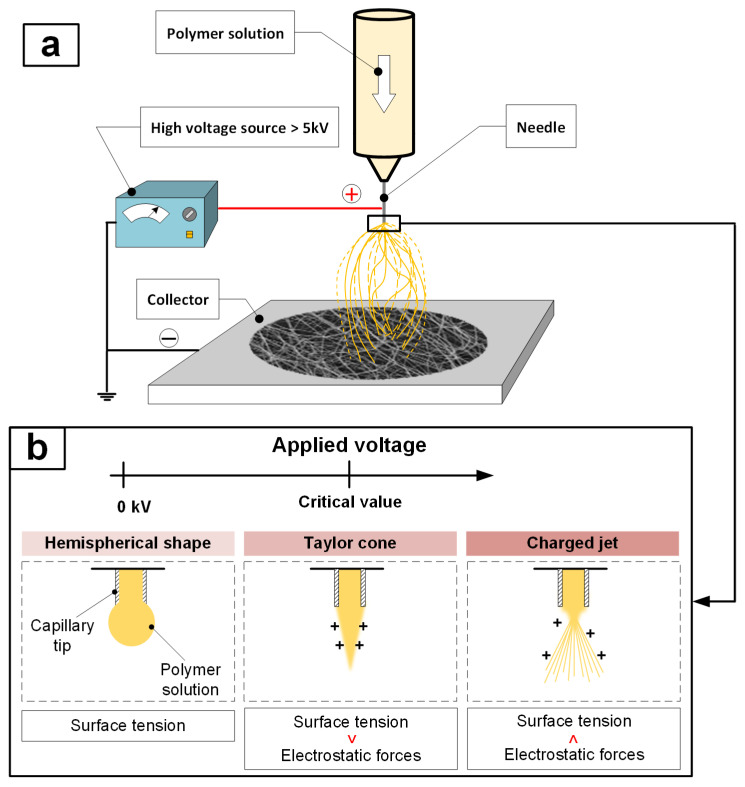
Electrospinning: diagram of the electrospinning device (**a**); principle of operation of electrospinning (**b**).

Electrospinning is one of the most widely used processes for obtaining nanofibers and nonwoven materials based on them [1,2]. The principle of electrospinning is based on the creation of a potential difference between the end of the needle through which the polymer solution is fed and a grounded collector through which the resulting micro- or nanofiber is deposited [3,4]. The potential difference generated by applying a high voltage to the end of a positively charged needle and a negatively charged collector forms an electric field between them (Figure 1a). The resulting electric field force causes the polymer solution to flow from the end of the needle as a so-called Taylor cone [5,6,7,8]. When the force of the electric field overcomes the surface tension of the polymer solution, a jet is formed at the end of a positively charged droplet, which, under the action of repulsive and instability forces, disintegrates into many thin polymer threads (Figure 1b), deposited on the negatively charged collector [9]. In this case, the volatile solvent evaporates during the process of deposition of fibers on the collector.

Currently, other methods for producing nanofibers without the use of high voltage are known, such as centrifugal spinning [10,11,12], melt blowing [13,14,15], and phase separation [16]. However, electrospinning is the most promising method, as it can be scaled up to produce homogeneous nanofiber materials in large quantities. For example, using a collector consisting of a system of rollers—between which a substrate is passed and onto which the fiber is deposited—increases the output of produced nonwoven materials from 10 to 800 m^2^/h [17].

In recent years, much attention has been paid to research aimed at producing CNFs, primarily due to their high specific tensile strength, high specific Young’s modulus, chemical, thermal and corrosion resistance, and lightweight [18,19]. CNFs are long, thin filaments with diameters ranging from 10 to 1000 nm, consisting mainly of carbon atoms bonded together in microscopic crystals and aligned parallel to the long axis of the fiber [20]. CNFs are widely used as reinforcement in composites, and their applications have extended from aerospace and military industries to modern industries such as automotive and energy [21,22].

This study focuses on the development of flame-retardant materials based on CNFs. Worldwide statistics show an annual increase in the frequency and intensity of industrial and forest fires. USA fire departments annually extinguish between 36,784 and 37,000 fires at industrial and manufacturing facilities [23]. The main causes of industrial and manufacturing fires are heating equipment, tools, and industrial equipment, each accounting for about 14% [24]. Fires in structures, which account for about 20% of all fires at these facilities, are the cause of the greatest property damage and a significant number of injuries and deaths among civilians. Faulty electrical equipment is the leading cause of workplace fires in the UK [25]. Forest fires are also increasing worldwide [26]. Therefore, the urgent task is to develop effective flame-retardant materials that will be useful in heat-protective clothing for firefighters, flame-retardant blankets and fabrics that will help extinguish and eliminate fires inside buildings and industrial facilities.

Carbon fibers can be produced from various polymer precursors: lignin, cellulose, polyimide (PI), pitch-based precursors, PAN, etc. In recent studies [27,28], lignin and cellulose were considered an alternative to PAN, which is most often used to produce CNFs. Lignin and cellulose are biodegradable polymers of natural origin and have a carbon-forming chemical structure, but the carbon yield is moderate and is 20–30% for lignin and 25–30% for cellulose, which is significantly lower than PAN. In addition, lignin- and cellulose-based fibers require additives that improve their thermal stability or mechanical properties. In [29], the production of porous PI-based CNFs, which have high thermal stability, mechanical strength, and flexibility, was reported. However, the study [30] indicates that aromatic PIs are insoluble in common organic solvents, while their precursors, such as polyamic acid, are soluble. Therefore, PI-based nanofibers are obtained in two stages: 1—electrospinning of the PI precursor, polyamic acid (PAA), in dimethylacetamide or dimethylformamide to obtain PAA nanofibers; 2—imidization of the obtained PAA nanofibers at high temperature to form PI fibers. Due to the presence of a number of intermediate stages in the production of PI nanofibers, this technology is expensive. The study [31] reported the use of pitch-based precursors: naphthalene is a monomer for the preparation of mesophase pitch, which can be obtained by thermal polymerization or chemical synthesis. It has a carbon yield of about 85%. The CNFs synthesized from this precursor show high modulus due to their dominant graphitic nature but exhibit poor compression compared to PAN-based CNFs. PAN is widely used among the listed precursors because it is commercially available, promotes the formation of stable fibrous structures during electrospinning, and provides a high carbon yield of about 56% [20,32], significantly higher than other polymer precursors. Also, PAN-based nanofibers exhibit a high tensile strength of 212 GPa [33], flexibility, and structural integrity.

At the same time, obtaining PAN fibers by electrospinning ensures a uniform distribution of fiber diameters, the size of which is in the nano range. In obtaining PAN-based CNFs, thermal stabilization of the original nanofiber in an air atmosphere is necessary [34,35]. Elevated temperature during stabilization initiates cyclization, oxidation and dehydrogenation reactions. As a result, linear PAN chains are transformed into a cyclic structure, which can keep the fibers infusible and nonflammable during further carbonization at higher temperatures [36]. Due to high thermal stability and low weight, PAN-based CNFs can be used in heat-protective clothing, spark protection in welding blankets, flame-retardant mattresses, and industrial seals. Often, to improve flame-retardant and mechanical properties, nanoparticles of various metals or metal oxides, for example, TiO_2_, ZnO, and Fe_3_O_4_, are added to the composition of nanofibers [37,38,39,40].

This work aimed to obtain PAN-based carbon nanofibers, as well as TiO_2_-modified PAN nanofibers, and their comprehensive study, including the dependence of fiber diameters on the viscosity of the initial solutions, the effect of temperature treatment on the functional groups of PAN, elemental analysis and assessment of the refractory properties of the developed nanofibers and CNFs. First, this work allowed us to identify certain patterns and select the concentrations of PAN and TiO_2_ suitable for obtaining CNFs. The PAN-based CNFs presented in this manuscript are not the final material ready for use. However, the work results will be useful for further developing multilayer heat-insulating and flame-retardant composite materials based on freeze-dried polycaprolactone modified by the PAN-based CNFs obtained in this work.

## 2. Materials and Methods

### 2.1. Materials and Reagents Used

The following chemical reagents and materials were used in the work. The synthetic polymer PAN with a molecular weight of 50 kDa (Hebei Chuanghai Biotechnology Co., Ltd., Shijiazhuang, China) was used as the fiber base, and chemically pure dimethyl sulfoxide (DMSO) with a content of the main substance of at least 99.9% (Kemstor, Moscow, Russia) was used as a solvent. DMSO was chosen as a solvent for several reasons. Firstly, DMSO is less carcinogenic and does not belong to the standard class of flammable or toxic substances, unlike the more common dimethylformamide (DMF) and dimethylacetamide (DMA). At the same time, DMF and DMA belong to the third hazard class, according to the UN classification (UN Hazard Class: 3). Secondly, the boiling point of DMSO is 189 °C, the boiling points of DMF and DMA are 153 °C and 166 °C, respectively, while the vapor pressure of DMF and DMA is higher than that of DMSO. This indicates a higher volatility of DMF and DMA compared to DMSO. During the electrospinning process, the use of more volatile solvents leads to hardening of the polymer in the needle and, accordingly, clogging of the needle, which is highly undesirable.

Some fiber samples were modified with titanium oxide nanoparticles (TiO_2_), produced by the method of the electric explosion of a titanium conductor in an oxygen-containing atmosphere (Highly pure substances, Moscow, Russia). The average size of TiO_2_ nanoparticles indicated in the specification was 49 nm, and the bulk density was 0.5–2 g/cm^3^.

### 2.2. Development of an Electrospinning Installation

**Figure 2:**
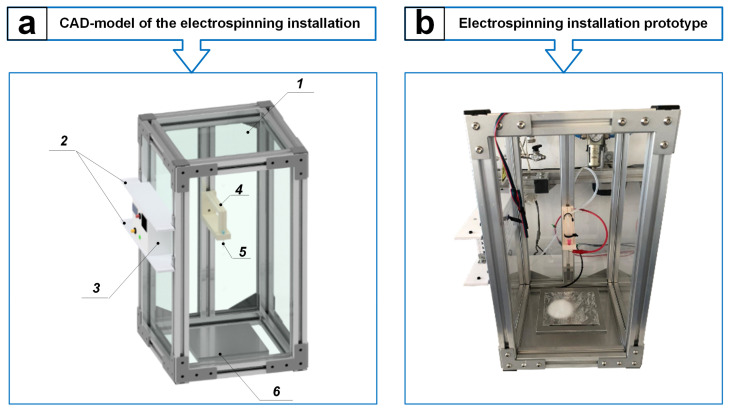
Electrospinning setup: CAD model (**a**), the setup body (1), PVC plates (2), control unit (3), needle mount (4), needle (5), plate-shaped collector (6); the appearance of the assembled setup (**b**).

An electrospinning setup of our own design was developed to obtain PAN carbon nanofibers. The development consisted of two main stages: 1—design of individual elements of the setup structure and assembly of a full-fledged three-dimensional CAD model (Figure 2a); 2—implementation of the electrospinning setup structure in metal according to the developed CAD model (Figure 2b).

The electrospinning setup (Figure 2) consists of a frame in the form of an elongated parallelepiped with overall dimensions of 480 × 220 × 220 mm, made of aluminum profiles connected to each other by L-shaped plates. Groove seals are inserted on the inside of the aluminum profiles, into which transparent polycarbonate glasses, 3 mm thick each, are installed. The described elements together form the housing of the setup (1). Two aluminum corners, located at a distance of 80 mm from each other, are bolted onto two aluminum profiles, which form the setup’s left side. In this case, polyvinyl chloride plates (2) with dimensions of 5 × 220 × 50 mm are attached between the corners at the same height, between which a control unit (3) of our own design is inserted. A central aluminum profile is installed on the rear side of the housing, on which the amount (4) for the needle (5) is fixed. In this case, the fastening (4) is installed in such a way that its distance to the collector (6) can be changed by loosening the bolts and moving it up or down along the central aluminum profile, and the outlet of the needle (5) is located exactly in the center of the base of the housing (1) of the installation above the collector (6) in the form of a plate.

The developed installation allows the electrospinning process to be organized in a single housing, and the plate-shaped collector can be replaced with a rotating disk or drum, which is regulated using the control unit (3).

The auxiliary elements of the electrospinning unit are a high-voltage source and a syringe pump. The high-voltage source of Chinese manufacture is presented in the form of a rectangular block with overall dimensions of 200 × 120 × 70 mm and is equipped with a ventilation system to prevent overheating of the device, as well as two insulated wires responsible for the positive and negative phases. This high-voltage source operates in the range from 2 to 16 kV. The syringe pump from SONO-TEK (Milton, NY, USA), with overall dimensions of 230 × 150 × 130 mm, can change the flow rate from 0.1 μL/h to 506 mL/h.

### 2.3. Methodology for Obtaining CNFs Based on PAN

**Figure 3:**
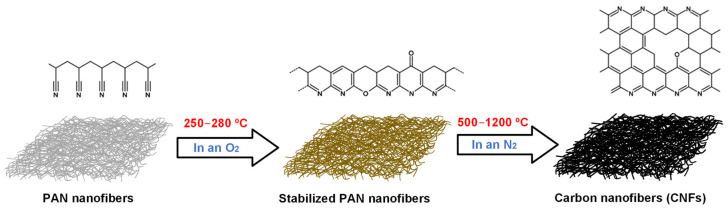
Scheme for obtaining CNFs based on PAN.

The obtaining of PAN-based CNFs was carried out in three main stages: 1—obtaining of PAN nanofibers by electrospinning; 2—heat treatment in an oxygen environment to produce stabilized PAN nanofibers; 3—treatment of stabilized PAN nanofibers in a nitrogen environment at temperatures above 500 °C to produce CNFs. Figure 3 shows the general scheme for producing PAN-based CNFs.

In the first stage of the work, PAN with three different concentrations of 13, 14 and 15 wt.% was dissolved in DMSO to prepare three solutions of 50 mL. Each solution was stirred on a magnetic stirrer with heating (T = 55 °C) for 2 h until a homogeneous light-yellow consistency was obtained. Additionally, three more solutions of 50 mL were prepared containing 15 wt.% PAN, to each of which TiO_2_ nanoparticles were added—1, 2 and 3 wt.%, respectively. Each solution with different TiO_2_ contents was homogenized on an ultrasonic homogenizer (Bandelin Sonopuls HD 2200, Berlin, Germany) for 20 min to distribute the nanoparticles throughout the solution volumes uniformly. All six solutions were used to obtain PAN nanofibers and PAN nanofibers modified with TiO_2_ nanoparticles. The nanofibers were obtained by electrospinning using the setup described in Section 2.2. The deposition process of PAN nanofibers is shown in Video S1. Table 1 presents the specifications of the obtained PAN nanofiber samples and the conditions for the electrospinning process.

In the second stage of the work, some of the previously obtained PAN nanofiber samples were subjected to heat treatment in an oxygen environment at 170 °C, 210 °C and 250 °C to study the effect of temperature on the stabilization of PAN nanofibers. Table 2 shows the specifications of these samples.

In the work’s third stage, PAN-based CNF samples were obtained by carbonizing previously stabilized at 250 °C nanofiber samples in an inert nitrogen atmosphere. PAN nanofiber carbonization was carried out in a muffle furnace at the Department of Inorganic Substances and Electrochemical Processes of the MUCTR. The heating rate of the muffle furnace was 5 °C/min and decreased as the set temperature approached 520 °C/min. The nitrogen consumption during the experiment was 0.2 L/min. Table 3 shows the specifications of the obtained PAN-based CNF samples.

The initial solutions described in this section and the obtained samples of nanofibers and CNFs based on PAN were studied using the methods given below.

### 2.4. Study of the Effect of Temperature Treatment of PAN Nanofibers on Their Resistance to DMSO

Four samples of the obtained fibers PAN-13, PAN-13-T170, PAN-13-T210, and PAN-13-T250 were tested for resistance to the initial solvent DMSO to indirectly confirm the stabilization of PAN. For this purpose, one drop of DMSO was added to the surface of the samples through a syringe and a timer was started. The appearance of the samples after adding DMSO was recorded after 1 min and after 1 h.

### 2.5. Infrared Spectroscopy (IR Spectroscopy)

To study the effect of temperature treatment on the vibrational spectra of molecules and functional groups in nanofiber samples PAN-13, PAN-13-T170, PAN-13-T250, and PAN-13-T250-T520, the IR spectroscopy method was used in the work. Sample PAN-13 (control) was not treated with temperature and was used to compare and determine the effect of temperature treatment on the vibrational spectra of molecules.

The study used a Nicolet 380 FTIR spectrometer (Thermo Fisher Scientific Inc., Waltham, MA, USA). The following spectral range was used in the work: 4000–500 cm^−1^. The analytical study was conducted at the MUCTR Shared Use Center.

### 2.6. Scanning Electron Microscopy (SEM)

A VEGA3-LMU TESCAN scanning electron microscope (TESCAN, Brno, Czech Republic) was used to visualize, study, analyze and compare the external structure of the PAN-13, PAN-14, PAN-15, PAN-15-T250, PAN-15-T250-T520, PAN-15-TiO_2_-3, PAN-15-TiO_2_-3-T250-T520 fiber samples. To prevent charge accumulation, 1 nm of gold was sputtered on the surface of the samples before scanning. The analytical study was conducted at the Department of Chemistry and Technology of Crystals MUCTR.

### 2.7. Study of Viscosity of Initial PAN Solutions

The viscosity study was carried out for six initial PAN solutions: PAN-13, PAN-14, PAN-15, PAN-15-TiO_2_-1, PAN-15-TiO_2_-2, PAN-15-TiO_2_-3 using an Anton Paar SmartPave 102e rotational rheometer (Anton Paar, Graz, Austria). The measurements were carried out at a constant shear rate of 0.1 s^−1^ and a temperature of 25 °C. The number of measurement points for each solution was 20. The analytical study was carried out at the Department of Chemical and Pharmaceutical Engineering MUCTR. The study allowed for further analysis of the dependence of the diameter of the formed PAN fibers on the viscosity of the initial solutions.

### 2.8. Elemental Analysis of the Obtained Samples (EDS Analysis)

Elemental analysis was performed for the samples modified with titanium oxide: PAN-15-TiO_2_-1, PAN-15-TiO_2_-2, PAN-15-TiO_2_-3, PAN-15-TiO_2_-1-T250-T520, PAN-15-TiO_2_-2-T250-T520, PAN-15-TiO_2_-3-T250-T520 using an X-ray spectral energy-dispersive microanalyzer (EDS Oxford Instruments X-MAX-50, High Wycombe, UK) based on a VEGA3-LMU TESCAN scanning electron microscope (TESCAN, Czech Republic). The analytical study was carried out at the Department of Chemistry and Technology of Crystals MUCTR.

### 2.9. Differential Scanning Calorimetry and Thermogravimetric Analysis

Differential scanning calorimetry (DSC) is a thermoanalytical technique used to monitor and measure the amount of heat absorbed or released by a sample as it is heated or cooled. DSC primarily provides data on transition temperatures, transition enthalpies, and the specific heat capacity of a particular material. This technique is particularly useful in determining changes such as melting point, glass transition, crystallization, and thermal stability.

Thermogravimetric analysis (TGA) is a technique that shows the change in mass of a sample as a function of heating temperature. TGA primarily provides information on compositional changes in a material, especially related to volatile components, moisture content, thermal stability, and decomposition patterns.

To perform DSC and TGA analysis and determine the thermal stability of the samples (PAN-15, PAN-15-T250-T520, PAN-15-TiO_2_-1, PAN-15-TiO_2_-2, PAN-15-TiO_2_-3, PAN-15-TiO_2_-1-T250-T520, PAN-15-TiO_2_-2-T250-T520, PAN-15-TiO_2_-3-T250-T520), an SDT Q-600 thermal analyzer (TA Instruments, New Castle, DE, USA) with a heating rate of 10 °C/min, from room temperature to 1000 °C. The measurements were carried out in a nitrogen atmosphere at a 20 mL/min flow rate. The study was carried out at the Department of Chemistry and Technology of Crystals MUCTR.

### 2.10. Visualization of Flammability of PAN Nanofibers

To visualize the difference in flame-retardant properties between the original PAN nanofiber (PAN-15), stabilized PAN nanofiber (PAN-15-T250) and PAN-based CNFs (PAN-15-T250-T520), video and photographic recording of the combustion process of these samples in air were performed.

## 3. Results and Discussion

### 3.1. Effect of Temperature Treatment of Nanofibers on the Stabilization of PAN

**Figure 4:**
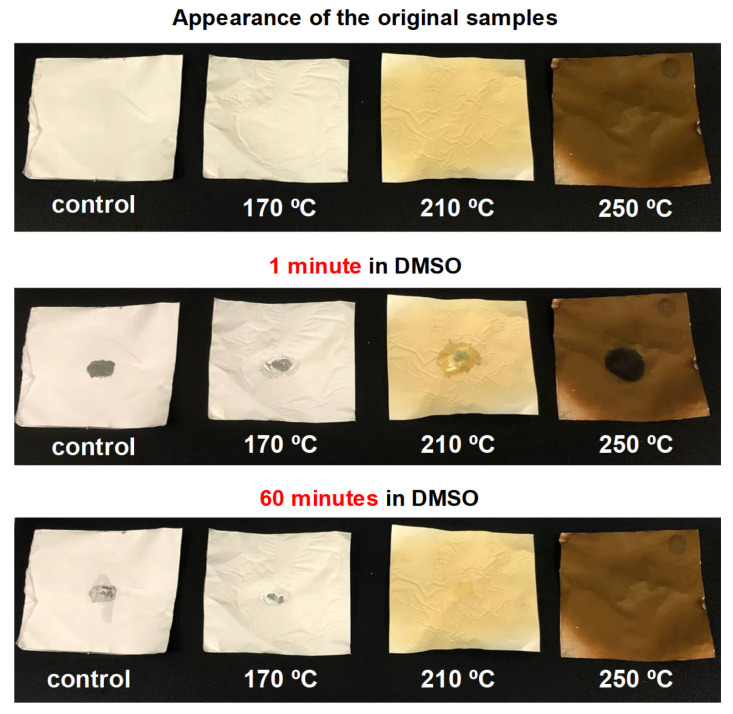
Study of samples for resistance to the initial solvent DMSO: from left to right PAN-13, PAN-13-T170, PAN-13-T210, PAN-13-T250.

The results of the study of the effect of temperature treatment of PAN nanofibers on their resistance to DMSO are presented in Figure 4 and also in Figures S1–S3.

**Figure 5:**
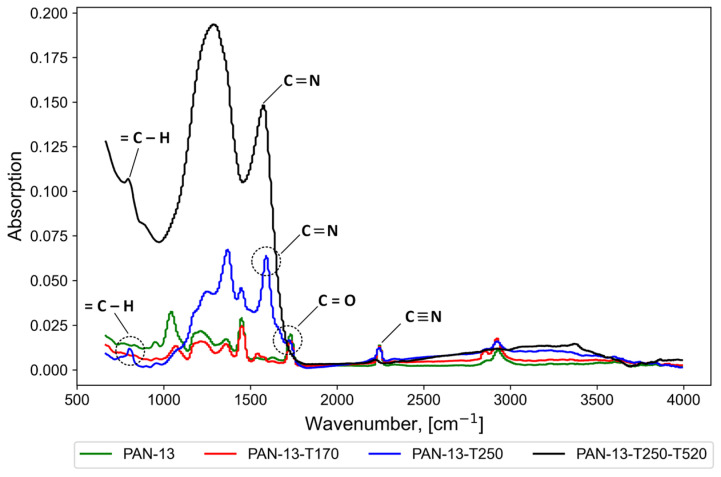
Results of IR spectroscopy of PAN nanofibers.

The top of Figure 4 shows the samples at the initial time before adding DMSO, then 1 min and 60 min after adding a drop of DMSO to the surface of the samples. It can be seen that the PAN sample treated at 250 °C (PAN-13-T250) is resistant to the effects of DMSO, which indirectly confirms the stabilization of polyacrylonitrile. Meanwhile, the fibers of the PAN-13, PAN-13-T170, and PAN-13-T210 samples dissolve under the action of DMSO. The change in the color of the samples from white to dark brown also indicates a chemical reaction during heat treatment. However, for more accurate conclusions about the effect of temperature on functional groups in the PAN-13, PAN-13-T170, PAN-13-T250 and PAN-13-T250-T520 nanofiber samples, IR spectroscopy was carried out, the results of which are shown in Figure 5.

The analysis (IR) showed vibrations of the nitrile group (C≡N)—peaks at 2242 cm^−1^ and 1451 cm^−1^, as well as carboxylic acid groups, including the (C=O) group at 1730 cm^−1^, the (C–O) and (O–H) groups at 1200 cm^−1^ [34,41]. The absorption band at 1589 cm^−1^ is mainly due to vibrations of the double bond (C=N), as well as to the combination of (C=N), (C=C), and (N–H) [36]. The peaks at 1350–1370 cm^−1^ are due to bending and stretching vibrations (CH_2_). The peaks at 1044–1070 cm^−1^ are due to ether vibrations (C–O–C) of comonomers such as itaconic acid or methyl acrylate, which are often used in the obtaining of PAN [42]. The peak at 800–807 cm^−1^ is attributed to aromatic vibrations (=C–H), which occur in the presence of oxygen as a result of aromatization, resulting in the removal of hydrogen in the form of H_2_O [36,42].

Thermal oxidation of PAN consists of two main stages: 1—cyclization and 2—dehydrogenation. At the cyclization stage, triple bonds (C≡N) are transformed into double bonds (C=N). Characteristic peaks (C=N) at 1589 cm^−1^ are observed for samples PAN-13-T250 and PAN-13-T250-T520. For samples PAN-13 and PAN-13-T170, the peaks in the absorption band at 1589 cm^−1^ are not observed. Therefore, the temperature of 170 °C is not enough to start the cyclization reaction. At the same time, triple bonds (C≡N) are still present in sample PAN-13-T250—the peak at 2242 cm^−1^. Therefore, the temperature of 250 °C is not enough to eliminate all triple bonds (C≡N). In the second stage, dehydrogenation leads to the formation of aromatic structures. During the dehydrogenation process, a peak at 800 cm^−1^ corresponding to the (=C–H) bonds appears, which is observed for samples PAN-13-T250 and PAN-13-T250-T520. For sample PAN-13-T170, the peak in the absorption band of 800 cm^−1^ is absent since the temperature of 170 °C is insufficient for the formation of aromatic structures. It should also be noted that the ether vibrations (C–O–C) are most pronounced for the original sample PAN-13 at 1044 cm^−1^. With an increase in temperature to 170 °C, the peak intensity decreases, which is seen in sample PAN-13-T170. With a further increase in temperature to 250 °C (sample PAN-13-T250), the peak characteristic of (C–O–C) disappears.

The obtained results showed that preliminary heat treatment at 250 °C promotes the stabilization of PAN nanofibers. The ongoing cyclization and dehydrogenation reactions promote the formation of a cyclic structure from a linear PAN molecule. The cyclic structure makes PAN more thermally stable and prevents its melting at the carbonization stage. Analysis of the IR spectra for the PAN-13-T250-T520 CNFs sample showed that the intensity of the peaks corresponding to the (C=N) and (=C–H) bonds increases significantly, and the peak corresponding to (C≡N) disappears.

### 3.2. Results of the Study of the Structure of PAN Nanofibers

**Figure 6:**
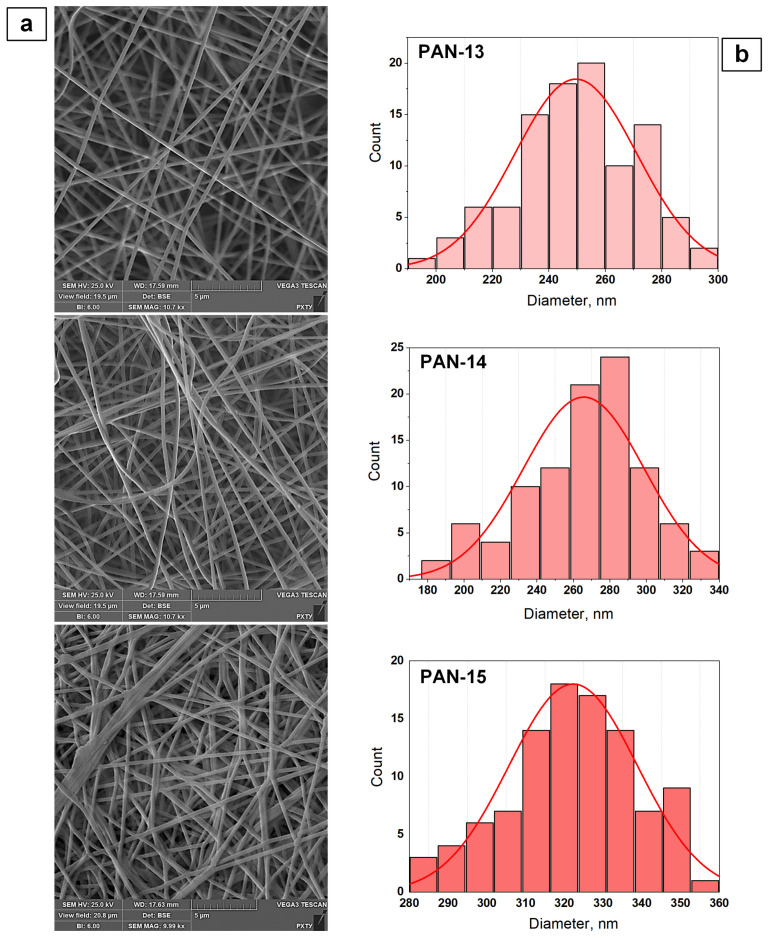
Effect of PAN concentration on the external structure of nanofibers: SEM images of samples PAN-13, PAN-14, PAN-15 (**a**); fiber diameter size distribution (**b**).

During the experimental work, SEM images were obtained for a number of initial nanofiber samples that differed in the concentration of PAN used: PAN-13, PAN-14, and PAN-15 (Figure 6a). Based on the results of processing the SEM images in the ImageJ (version 1.53k) program, fiber diameter distributions by size were constructed (Figure 6b).

Analysis of the obtained results (Figure 6) showed that with increasing PAN concentration, the fiber diameter gradually increases. For samples PAN-13, PAN-14 and PAN-15, the average number diameters of fibers were calculated using Equation (1):

(1) $${\bar{d}}_{n}=\sum_{i}\frac{n_{i}}{\sum_{i}n_{i}}d_{i},$$

where ${\bar{d}}_{n}$—the average diameter, nm; *n_i_*—the number of fibers in the *i*-th fraction; *d_i_*—the diameter of fibers in the *i*-th fraction, nm; $\sum_{i}n_{i}$—the total number of measured fibers; $\frac{n_{i}}{\sum_{i}n_{i}}$—the numerical proportion of fibers in the *i*-th fraction.

**Figure 7:**
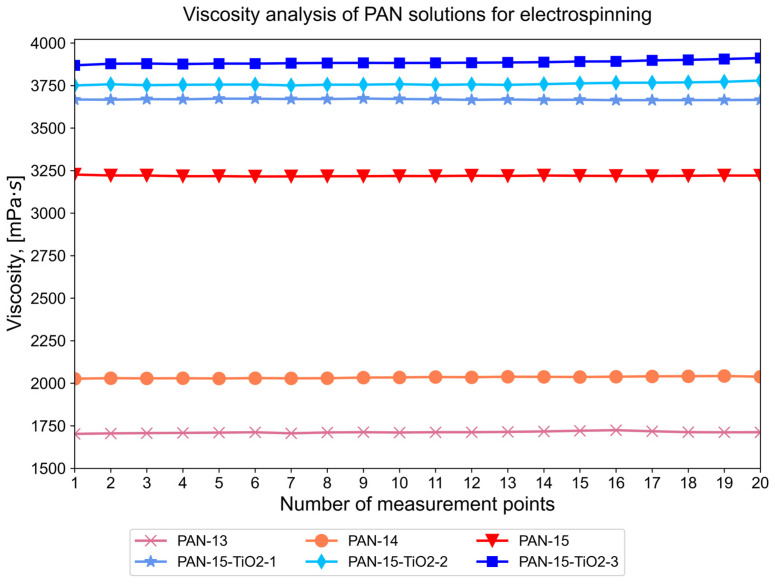
The viscosity of initial PAN solutions of different concentrations and PAN solutions modified with TiO_2_ with different mass contents.

The calculated number-average diameters of nanofibers for samples PAN-13, PAN-14, and PAN-15 were 250, 266.4, and 322.3 nm, respectively. It can be seen that the number-average diameter for sample PAN-14 increases by 16.4 nm compared to PAN-13, while for sample PAN-15, the number-average diameter sharply increases by 55.9 nm compared to PAN-14. Consequently, the diameters of nanofibers increase nonlinearly, which is primarily due to the nonlinear increase in the viscosity of the initial solutions of PAN-13, PAN-14, and PAN-15, depending on their concentration (Figure 7).

The nonlinear increase in the viscosity of PAN-13, PAN-14 and PAN-15 solutions with an increase in the PAN concentration with a step of 1 wt.% is associated with an increase in the prevalence of intermolecular interaction forces. At a certain concentration, the regions of polymer molecules begin to overlap, which leads to the entanglement of polymer chains and, as a consequence, to difficulty in flow. The results obtained are consistent with the data from the literature [43,44]. In this case, difficulty in the flow of the PAN solution jet during the electrospinning process leads to the deposition of fibers with a larger diameter. The viscosity value for the PAN-14 solution increases by 322.4 mPa s compared to the PAN-13 solution, while for the PAN-15 solution, compared to PAN-14, the viscosity sharply increases by 1184.6 mPa s.

**Figure 8:**
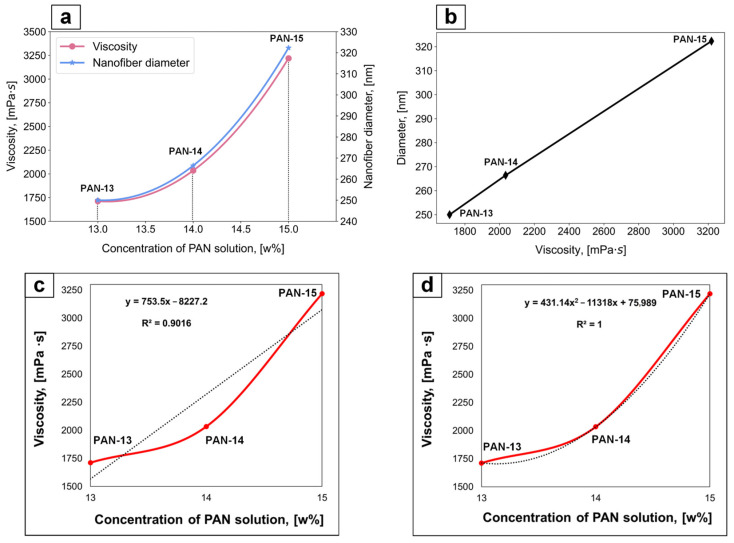
The nonlinear dependence of fiber diameter and solution viscosity on PAN concentration (**a**); linear dependence of fiber diameter on the viscosity of the corresponding PAN solutions (**b**); linear approximation of viscosity values (**c**); polynomial approximation of viscosity values (**d**).

The nonlinear increase in the fiber diameter and viscosity of PAN-13, PAN-14, and PAN-15 solutions with an increase in PAN concentration in 1 wt.% increment is shown in Figure 8a. It should be noted that the fiber diameter increases linearly with an increase in the viscosity of the corresponding solutions (Figure 8b).

The experimental data were approximated to confirm the nonlinear dependence of viscosity on concentration. With a linear approximation of viscosity values (Figure 8c), the determination coefficient (R^2^) is 0.9, while with an approximation of viscosity values by a second-order polynomial (Figure 8d), R^2^ is 1. If R^2^ approaches 1, this indicates a high degree of compliance of the experimental data with the model, while low values indicate that the model explains the dependencies poorly. The polynomial dependence describes the viscosity values better than the linear one. Graphs with the approximation of the fiber diameter values from the concentration of the initial PAN solutions are shown in Figure S4.

It should be noted that increasing the viscosity of PAN-13, PAN-14, and PAN-15 solutions leads to an increase in the number of nanofibers obtained per unit surface area (Figure 6a). For the PAN-15 sample, the SEM images show the effect of fiber aggregation.

From the presented results for samples PAN-13, PAN-14 and PAN-15, it can be concluded that the external structure and diameter of the formed fibers are strongly dependent on the viscosity of the initial solutions since the diameter of the fibers increases linearly with increasing viscosity of the corresponding solutions (Figure 8b). Therefore, the flow properties of the polymer solutions should be thoroughly investigated before carrying out the electrospinning process.

**Figure 9:**
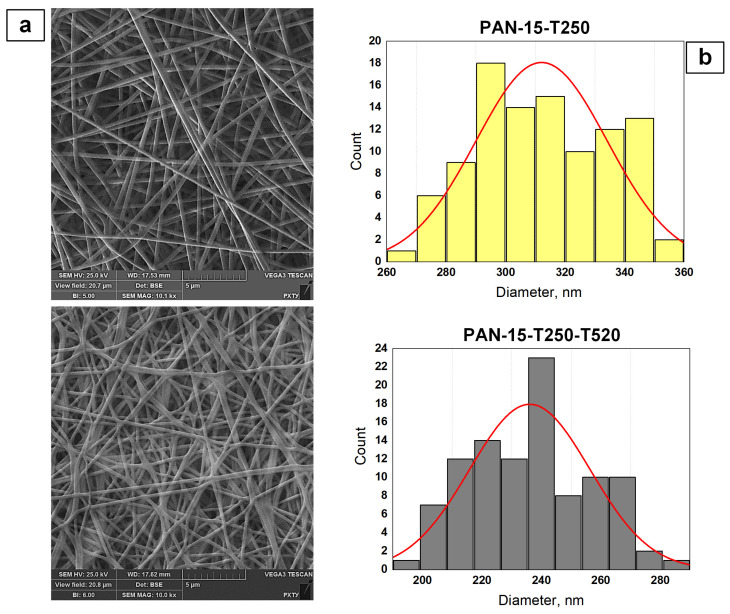
Effect of temperature treatment on the external structure of nanofibers: SEM images of PAN-15-T250 and PAN-15-T250-T520 samples (**a**); fiber diameter size distribution (**b**).

In subsequent experiments on obtaining PAN-based CNFs (Table 3), the PAN-15 sample was taken as a basis since the high viscosity of the solution increased the density of the deposited nanofiber on the collector per unit time. In this work, SEM images were obtained for the PAN-15-T250 nanofiber sample stabilized at a temperature of 250 °C and for the CNF sample calcined at a temperature of 520 °C, PAN-15-T250-T520 (Figure 9a).

The analysis of SEM images showed that the temperature treatment at 250 °C of the PAN-15-T250 sample had almost no effect on the nanofiber diameter. In comparison, the number-average diameter of fibers for PAN-15-T250 was 312.5 nm (Figure 9b), while for the original PAN-15 sample, the number-average diameter was 322.3 nm. Further, an increase in temperature treatment at 520 °C to obtain PAN-based CNFs led to thinning of the fibers of the PAN-15-T250-T520 sample. The number-average diameter for this sample decreased to 234.6 nm.

**Figure 10:**
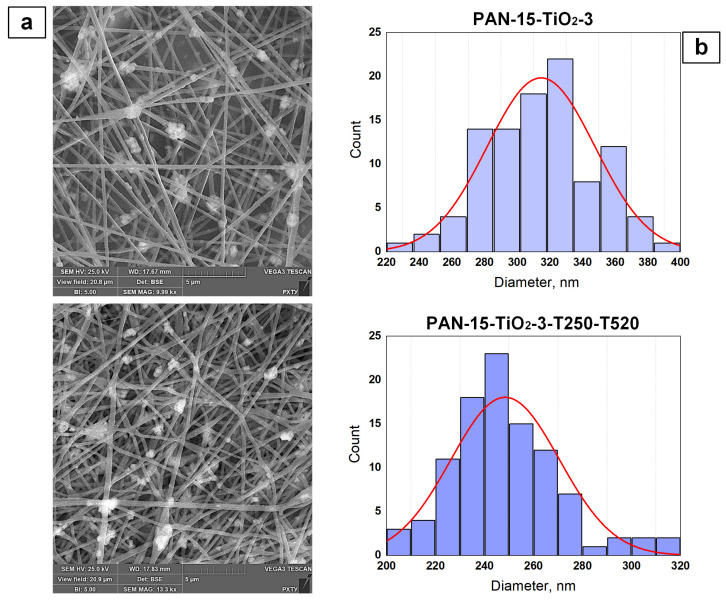
Effect of TiO_2_ additive on the external structure of nanofibers: SEM images of PAN-15-TiO_2_-3 and PAN-15-TiO_2_-3-T250-T520 samples (**a**); fiber diameter size distributions (**b**).

To study the effect of TiO_2_ additive on the external structure of nanofibers, SEM images were obtained for the original TiO_2_-modified nanofibers (PAN-15-TiO_2_-3) and for the TiO_2_-modified CNFs (PAN-15-TiO_2_-3-T250-T520). The SEM images are shown in Figure 10a.

The average diameter for the PAN-15-TiO_2_-3 sample was 314.6 nm, while for the PAN-15-TiO_2_-3-T250-T520 sample, the value was 248.6 nm. The obtained data on the number average diameter (Figure 10b) also confirm that the temperature treatment at 520 °C to obtain CNFs leads to thinning of the fibers. The addition of TiO_2_ nanoparticles to the initial solutions led to a significant increase in their viscosity (Figure 7). The solution for the PAN-15-TiO_2_-3 sample had the highest viscosity of 3886.4 mPa s. At the same time, the number average diameter of the fibers of the PAN-15 and PAN-15-TiO_2_-3 samples is almost the same (322.3 and 314.6 nm, respectively). In the case of the PAN-15-TiO_2_-3 sample, the viscosity does not affect the fiber diameter since its increase is associated only with the addition of TiO_2_ nanoparticles to the system. At the same time, the intermolecular interaction forces in the PAN-15-TiO_2_-3 solution remain the same as in the PAN-15 solution since the PAN concentration did not change. It should be noted that an increase in the mass concentration of TiO_2_ nanoparticles in the PAN solution leads to a linear increase in the viscosity of the solutions (Figure 7). The SEM images (Figure 10a) show the presence of TiO_2_ nanoparticle agglomerates. The formation of TiO_2_ agglomerates is also observed in the images of PAN-15-TiO_2_-1 (Figure S5), PAN-15-TiO_2_-1-T250-T520 (Figure S6), PAN-15-TiO_2_-2 (Figure S7), and PAN-15-TiO_2_-2-T250-T520 (Figure S8) samples. The formation of agglomerates is an undesirable process and is primarily associated with the homogenization parameters of the solutions (time and ultrasonic frequency of treatment).

### 3.3. Results of Elemental Analysis of PAN Nanofiber Samples

**Figure 11:**
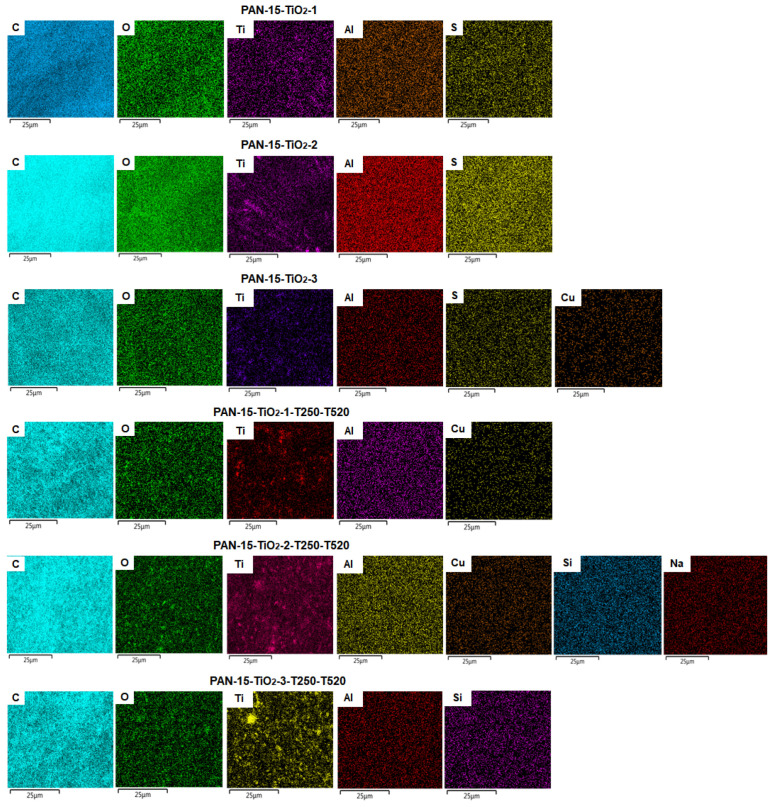
Surface mapping of nanofibers and CNFs.

Elemental analysis was performed for all TiO_2_-modified nanofiber and CNF samples. Figure 11 shows the surface mapping of the samples, reflecting the content of certain elements.

The numerical indices of the presence of elements in the samples of nanofibers and CNFs based on PAN modified with TiO_2_ are given in Table 4.

Surface mapping of the samples showed a minor presence of elements such as Al, S, Cu, Si and Na (Figure 11). The presence of Al, Cu, and Si in some samples is due to the fact that the nanofibers were deposited on the foil, and during the elemental analysis, the nanofibers were placed on the foil substrate. Aluminum foil contains small amounts of Cu and Si impurities. The presence of S in the PAN-15-TiO_2_-1, PAN-15-TiO_2_-2 and PAN-15-TiO_2_-3 samples is due to the dissolution of PAN in dimethyl sulfoxide (DMSO) before obtaining the nanofibers, but the remaining S disappears after calcining the samples at 520 °C. The presence of Na is insignificant.

The analysis of the results presented in Table 4 showed that the C content in the samples was higher than 80%. The highest weight value of C was observed in the PAN-15-TiO_2_-1-T250-T520 CNFs sample. In the PAN-15-TiO_2_-1, PAN-15-TiO_2_-2 and PAN-15-TiO_2_-3 nanofiber samples, as well as in the PAN-15-TiO_2_-1-T250-T520, PAN-15-TiO_2_-1-T250-T520 and PAN-15-TiO_2_-1-T250-T520 CNFs samples, the weight content of O increased, which was associated with an increase in the mass concentration of TiO_2_ in these samples. The highest weight content of Ti is observed in the CNF sample PAN-15-TiO_2_-2-T250-T520, which is associated with the calcination of TiO_2_ at 520 °C.

### 3.4. Characteristics of DSC and TGA for PAN Nanofibers

**Figure 12:**
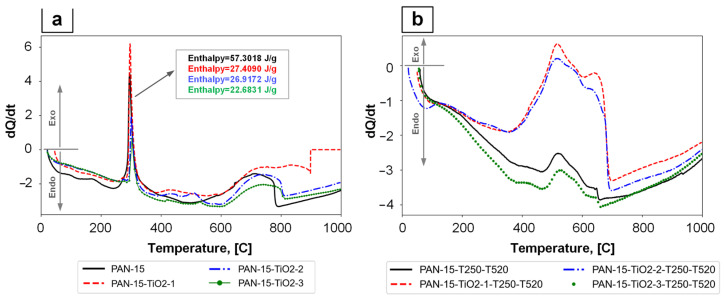
DSC results: PAN nanofiber samples of different compositions (**a**), PAN-based CNFs samples of different compositions (**b**).

DSC measures the heat flow associated with phase transitions and reactions as a function of temperature and time. The DSC results are shown in Figure 12.

During the DSC, the studied samples were heated from room temperature to 1000 °C. The heating of the samples was accompanied by two successive physicochemical processes—decomposition and combustion. First, the material decomposes (pyrolysis), which requires heat (endothermic process). Then, the decomposition products burn, during which heat is released (exothermic process). This heat (partially) is used to maintain decomposition. According to [45,46], the thermal effects Q_1_ (endothermic effect) and Q_2_ (exothermic effect) are of great importance. To be flame retardant, the material should have a high Q_1_ value (Figure 12—Endo) and a low Q_2_ value (Figure 12—Exo). As can be seen from the presented DSC results, for the original PAN nanofibers of different compositions (Figure 12a), characteristic peaks are observed, indicating an exothermic process in the temperature range of 290–320 °C. In this case, the peak height decreases with increasing TiO_2_ concentration (PAN-15-TiO_2_-1, PAN-15-TiO_2_-2, PAN-15-TiO_2_-3). For CNF samples of different compositions (Figure 12b) in the temperature range of 400–700 °C, the endothermic effect prevails, which may indicate the flame-retardant properties of these materials.

**Figure 13:**
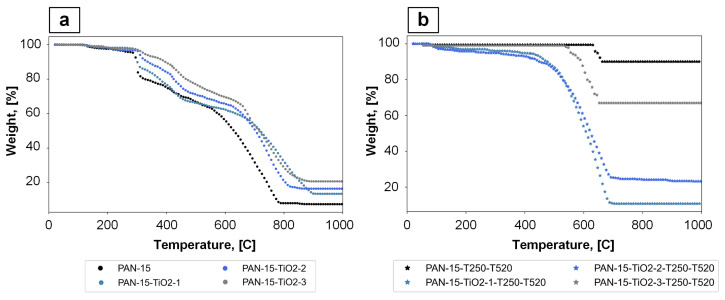
Results of TGA analysis: samples of PAN nanofibers of different compositions (**a**), samples of CNFs based on PAN of different compositions (**b**).

The thermal stability of the PAN nanofiber samples was investigated using TGA measurements in a nitrogen atmosphere. The TGA results are shown in Figure 13.

Based on the obtained TGA data, the LOI can be calculated. According to [45,46], there is a correlation between the material residue (SR) after temperature exposure and the LOI of polymers. This dependence is linear and can be represented by Equation (2):

(2) LOI=17.5+0.4·SR,

where *SR* is the sample residue at a temperature of 1000 °C, %.

The results of LOI calculation based on TGA analysis data are presented in Table 5.

The material should be considered flammable while the LOI value is <26%. Based on the results presented in Table 5, it can be concluded that the CNFs samples, namely PAN-15-T250-T520, PAN-15-TiO_2_-2-T250-T520, PAN-15-TiO_2_-3-T250-T520, have flame-retardant properties. At the same time, the highest LOI values are found in samples PAN-15-T250-T520 and PAN-15-TiO_2_-3-T250-T520 from this series. Lower LOI values for PAN-15-TiO_2_-1-T250-T520 and PAN-15-TiO_2_-2-T250-T520 samples may be due to the formation of agglomerates and uneven distribution of TiO_2_ nanoparticles in nanofibers, compared to PAN-15-TiO_2_-3-T250-T520 sample. At the same time, the LOI value for PAN-15-T250-T520 (without added TiO_2_) is higher than that of PAN-15-TiO_2_-3-T250-T520 sample, which may also indicate the uneven distribution of TiO_2_ nanoparticles in the sample. Therefore, future work is planned to study the effect of homogenization parameters on the uniformity of TiO_2_ nanoparticle distribution in nanofibers and determine the effect of agglomerate formation on the flame-retardant properties of the samples.

The calculated LOI values in this work are comparable with the LOI values for PAN-based fibers given in [36].

### 3.5. Results of Visual Comparison of Flammability of PAN Nanofibers

**Figure 14:**
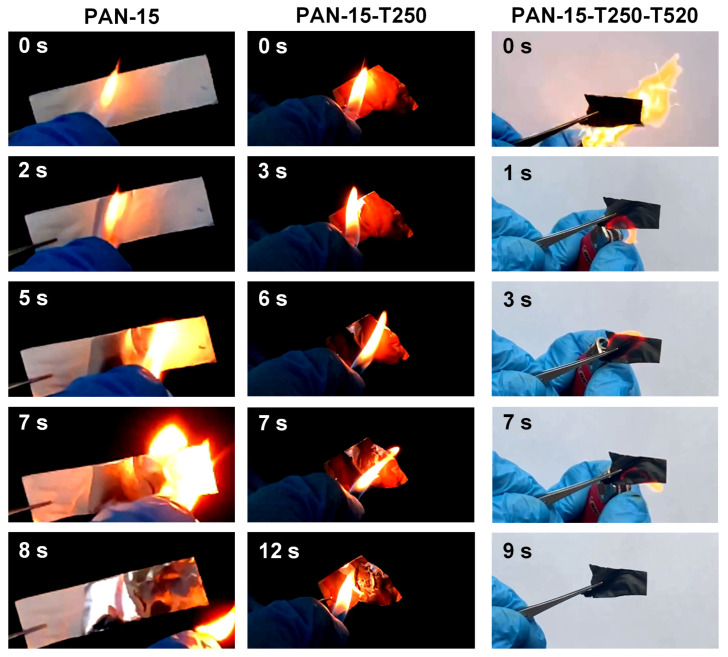
Visual comparison of the combustion process of PAN-15 (original nanofiber), PAN-15-T250 (stabilized nanofiber) and PAN-15-T250-T520 (CNFs) samples.

For a visual comparison of flame-retardant properties of the initial nanofibers, stabilized nanofibers and CNFs, samples PAN-15, PAN-15-T250 and PAN-15-T250-T520 were ignited with a lighter (the temperature at the top of the flame reaches 1200 °C) and photo and video recording of the combustion process was carried out. During the experiment, samples PAN-15 and PAN-15-T250 were on aluminum foil, and sample PAN-15-T250-T520 was ignited without foil. The results of the visual comparison of the combustion process of samples PAN-15, PAN-15-T250 and PAN-15-T250-T520 are presented in Figure 14.

The complete video sequence for the PAN-15, PAN-15-T250 and PAN-15-T250-T520 samples is available in Supplementary Materials—Video S2, Video S3 and Video S4, respectively.

The results showed that the sample of the original PAN-15 nanofibers begins to burn actively when an ignition source is brought near. The PAN-15-T250 sample gradually collapses and melts under the action of an open flame. The PAN-15-T250-T520 CNFs sample exhibits flame-retardant properties, does not burn, does not melt and does not deform. The obtained results are consistent with the results of the TGA analysis. And the calculated LOI. Therefore, the CNFs based on PAN obtained in the work can be recommended in the future for use in heat-protective clothing or other areas where the flame retardant property is key.

## 4. Conclusions

The electrospinning installation developed at the Mendeleev University of Chemical Technology of Russia made it possible to obtain PAN nanofibers. The subsequent development of a method for obtaining CNFs based on PAN made it possible to modify these nanofibers and give them flame-retardant properties.

The conducted comprehensive study of the characteristics obtained in the work of nanofibers and CNFs based on PAN showed that the diameter of the fibers and their external structure strongly depends on the viscosity of the initial solutions, an increase in viscosity leads to a linear increase in the diameter of the fibers. Preliminary temperature treatment at 250 °C helps to stabilize PAN nanofibers. The ongoing cyclization and dehydrogenation reactions contribute to the creation of a cyclic structure from a linear PAN molecule. This structure makes PAN more thermally stable and prevents its melting at the carbonization stage. Temperature treatment at 250 °C does not affect the diameter of the nanofiber. However, a subsequent increase in temperature to 520 °C leads to thinning of the fibers in the CNFs. The addition of TiO_2_ nanoparticles leads to a linear increase in the viscosity of PAN solutions, but this does not affect the diameter of the PAN nanofibers. Elemental analysis showed the weight content of carbon in the nanofiber samples is above 80%. The conducted flame retardant studies confirmed the flame retardant properties of the obtained CNF samples of different compositions. The LOI values calculated based on the TGA analysis results showed that the PAN-15-T250-T520, PAN-15-TiO_2_-2-T250-T520, PAN-15-TiO_2_-3-T250-T520 samples have flame retardant properties.

The developed PAN-based CNFs can potentially be used in applications where flame retardant is a key property, such as heat-protective clothing for firefighters, flame-retardant mattresses or blankets, and fire-retardant suits for the military.

This work is the first stage, and its results will form the basis for the multilayer heat-insulating and flame-retardant composite materials developed by the authors of the article based on lyophilized polymers (polycaprolactone and others), modified with the CNFs obtained in this work based on PAN.

## 5. Patents

SOFT, No. 2024687234: «Program for selecting and setting the operating mode of the stepper motor shaft in an electrospinning installation». Authors: Elizaveta Mokhova, Mariia Gordienko, Natalia Menshutina. Date of receipt: 15 November 2024.

## Figures and Tables

**Table 1:** Specification of the obtained PAN nanofiber samples.

Sample	Compound		Distance from Needle to Collector, mm	Flow Rate, mL/h	*V*, kV
	PAN, %	TiO_2_, %			
PAN-13	13	–	15	0.07	15
PAN-14	14	–			
PAN-15	15	–			
PAN-15-TiO_2_-1	15	1			
PAN-15-TiO_2_-2	15	2			
PAN-15-TiO_2_-3	15	3			

**Table 2:** Specification of PAN nanofiber samples subjected to preliminary temperature treatment.

Sample	Compound		Temperature Treatment, °C	Time, min
	PAN, %	TiO_2_, %		
PAN-13-T170	13	–	170	20
PAN-13-T210		–	210	
PAN-13-T250		–	250	
PAN-15-T250	15	–		

**Table 3:** Specification of PAN-based CNF samples.

Sample	Compound		Temperature Treatment, °C	Time, min
	PAN, %	TiO_2_, %		
PAN-13-T250-T520	13	–	520	60
PAN-15-T250-T520	15	–		
PAN-15-TiO_2_-1-T250-T520	15	1		
PAN-15-TiO_2_-2-T250-T520	15	2		
PAN-15-TiO_2_-3-T250-T520	15	3		

**Table 4:** Results of EDS analysis for TiO_2_-modified nanofiber and CNF samples.

Elements	C	O	Ti	Al	S	Cu	Si	Na
Sample	PAN-15-TiO_2_-1							
w., %	83.07	16.21	0.21	0.36	0.15	–	–	–
σ w.%	0.09	0.09	0.01	0.01	0.01	–	–	–
Sample	PAN-15-TiO_2_-2							
w., %	82.81	16.82	0.14	0.18	0.05	–	–	–
σ w.%	0.05	0.05	0.00	0.00	0.00	–	–	–
Sample	PAN-15-TiO_2_-3							
w., %	81.45	17.96	0.30	0.11	0.07	0.10	–	–
σ w.%	0.10	0.10	0.01	0.01	0.01	0.01	–	–
Sample	PAN-15-TiO_2_-1-T250-T520							
w., %	86.78	12.06	0.96	0.09	–	0.11	–	–
σ w.%	0.10	0.10	0.01	0.01	–	0.01	–	–
Sample	PAN-15-TiO_2_-2-T250-T520							
w., %	83.21	12.45	4.14	0.08	–	0.12	–	–
σ w.%	0.08	0.08	0.01	0.01	–	0.01	–	–
Sample	PAN-15-TiO_2_-3-T250-T520							
w., %	82.27	16.02	1.50	0.19	–	–	0.03	–
σ w.%	0.11	0.11	0.01	0.01	–	–	0.01	–

**Table 5:** Results of calculation of LOI.

Sample	SR, %	LOI, %
PAN-15	7.45	20.48
PAN-15-TiO_2_-1	13.48	22.89
PAN-15-TiO_2_-2	16.44	24.08
PAN-15-TiO_2_-3	20.68	25.77
PAN-15-T250-T520	90.05	53.52
PAN-15-TiO_2_-1-T250-T520	10.73	21.79
PAN-15-TiO_2_-2-T250-T520	23.25	26.80
PAN-15-TiO_2_-3-T250-T520	66.96	44.28

## Data Availability

Data are contained within the article and Supplementary Materials.

## Supplementary Materials

The following supporting information can be downloaded at https://www.mdpi.com/article/10.3390/polym17091255/s1. Figure S1: Image of samples before adding DMSO; Figure S2: Image of samples in 1 min after addition of DMSO; Figure S3: Image of samples in 60 min after addition of DMSO; Figure S4: Linear approximation of fiber diameter values from concentration (a), polynomial approximation of fiber diameter values from concentration (b); Figure S5: Microscopy of sample PAN-15-TiO_2_-1; Figure S6: Microscopy of sample PAN-15-TiO_2_-1-T250-T520; Figure S7: Microscopy of sample PAN-15-TiO_2_-2; Figure S8: Microscopy of sample PAN-15-TiO_2_-2-T250-T520; Video S1: deposition process of PAN nanofibers; Video S2: combustion study of the original PAN-15 sample; Video S3: combustion study of the stabilized PAN-15-T250 sample; Video S4: combustion study of the CNF PAN-15-T250-T520 sample.

## Abbreviations

The following abbreviations are used in this manuscript:

MUCTR	Mendeleev University of Chemical Technology of Russia
PAN	Polyacrylonitrile
CNFs	Carbon nanofibers
LOI	Limiting oxygen index
PI	Polyimide
PAA	Polyamic acid
DMSO	Dimethyl sulfoxide
DMF	Dimethylformamide
DMA	Dimethylacetamide
CAD	Computer-aided design
V	Voltage
PVC	Polyvinyl chloride
IR	Infrared spectroscopy
SEM	Scanning Electron Microscopy
EDS	Energy-dispersive X-ray spectroscopy
DSC	Differential scanning calorimetry
TGA	Thermogravimetric analysis
