# Supplementary figures and images for: Obtaining Polyacrylonitrile Carbon Nanofibers by Electrospinning for Their Application as Flame-Retardant Materials
Source: Polymers (Basel). 2025 May 5;17(9):1255. doi: 10.3390/polym17091255 (PMC12073878; doi:10.3390/polym17091255)

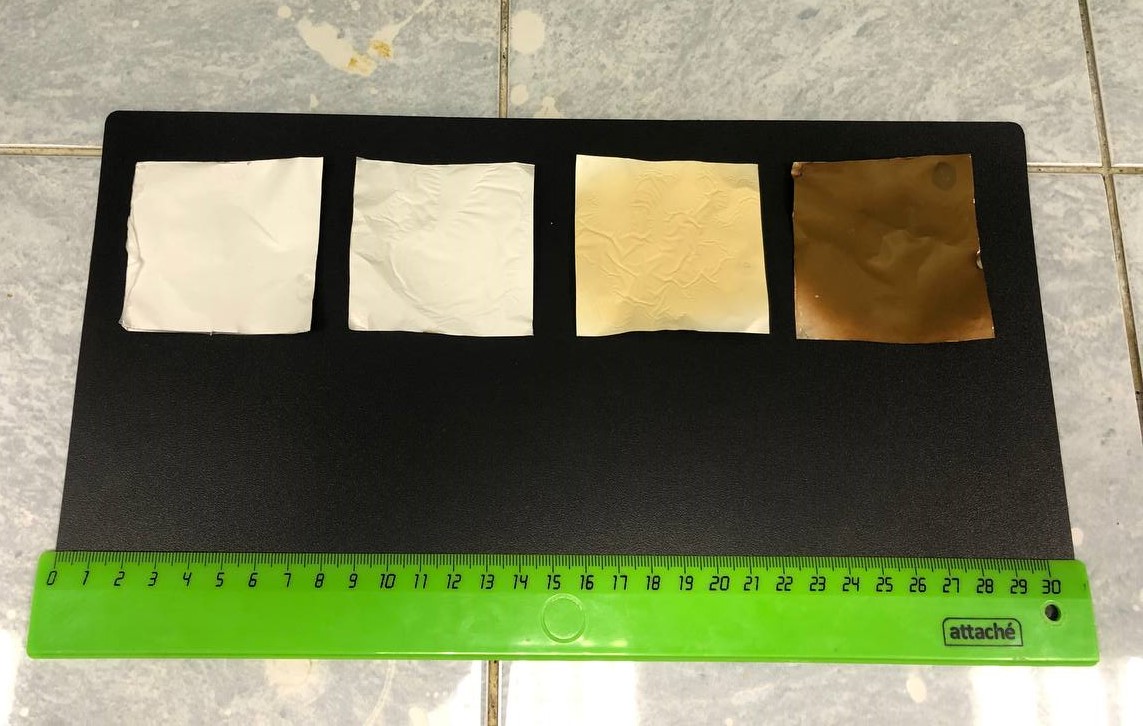

Supplement: Supplementary file 1 [file polymers-17-01255-s001.zip › Figure_S1.jpg]

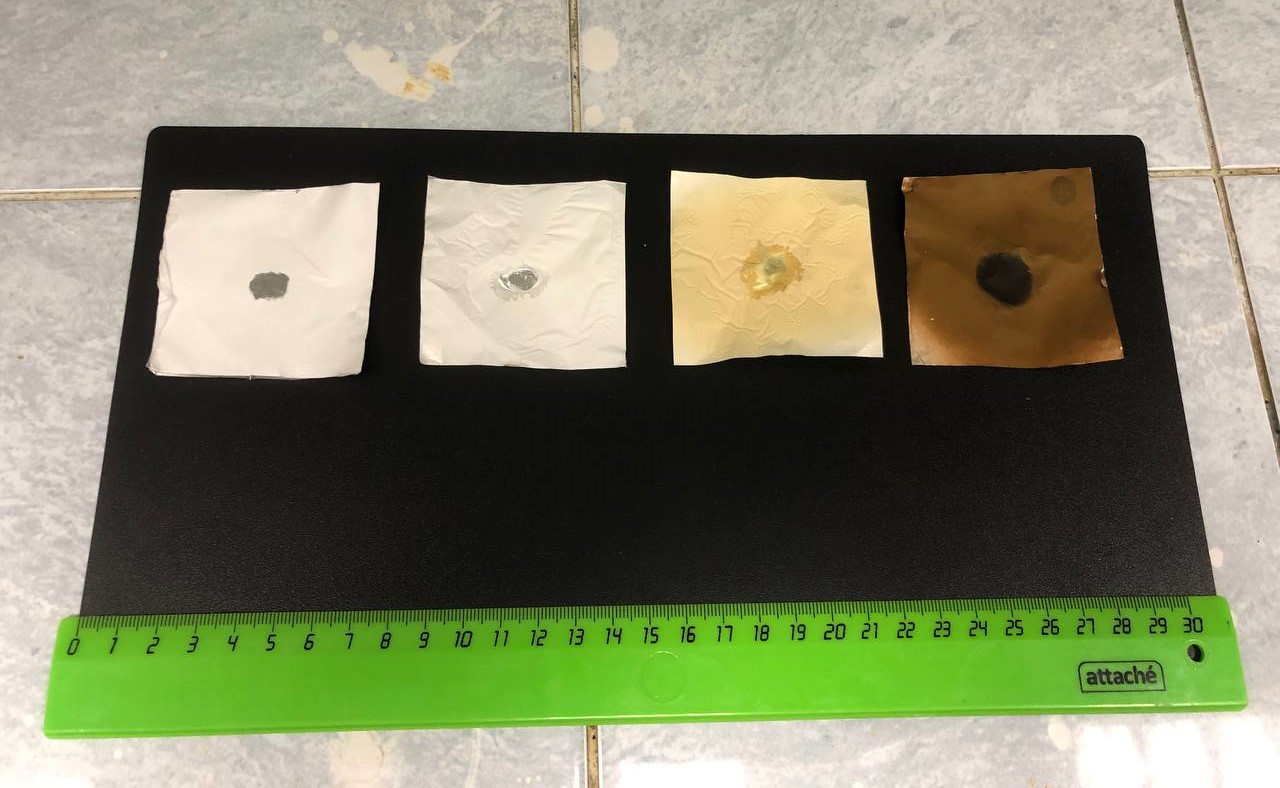

Supplement: Supplementary file 1 [file polymers-17-01255-s001.zip › Figure_S2.jpg]

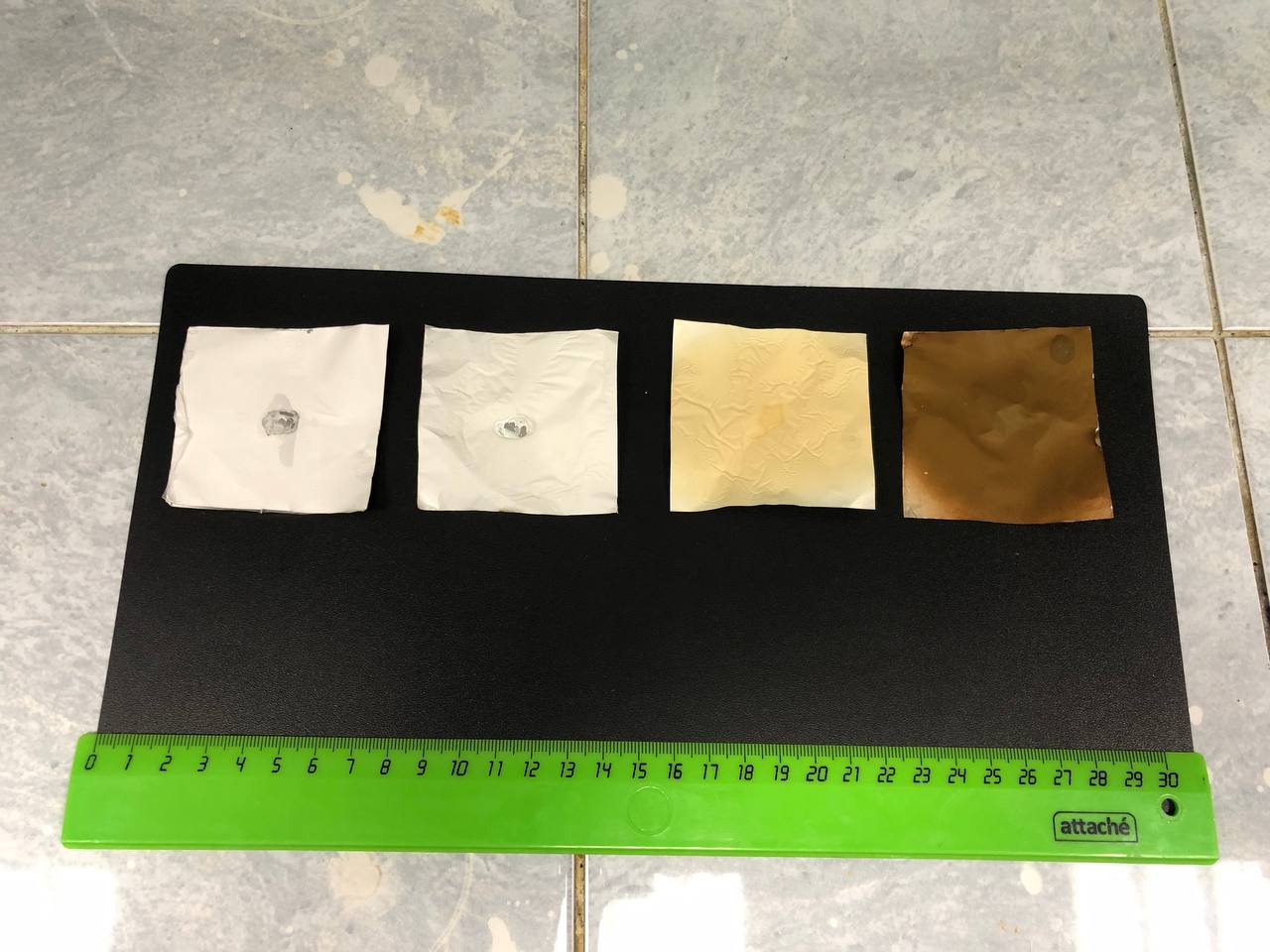

Supplement: Supplementary file 1 [file polymers-17-01255-s001.zip › Figure_S3.jpg]

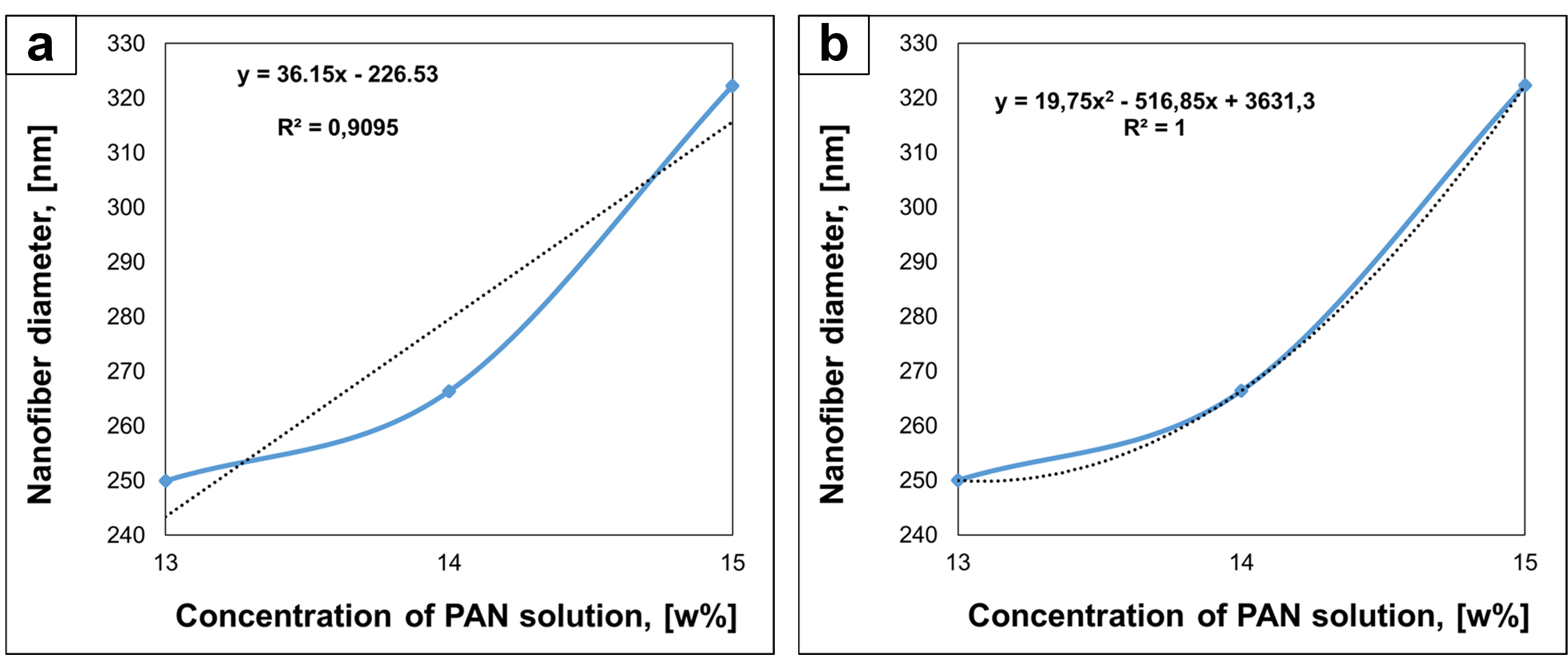

Supplement: Supplementary file 1 [file polymers-17-01255-s001.zip › Figure_S4.png]

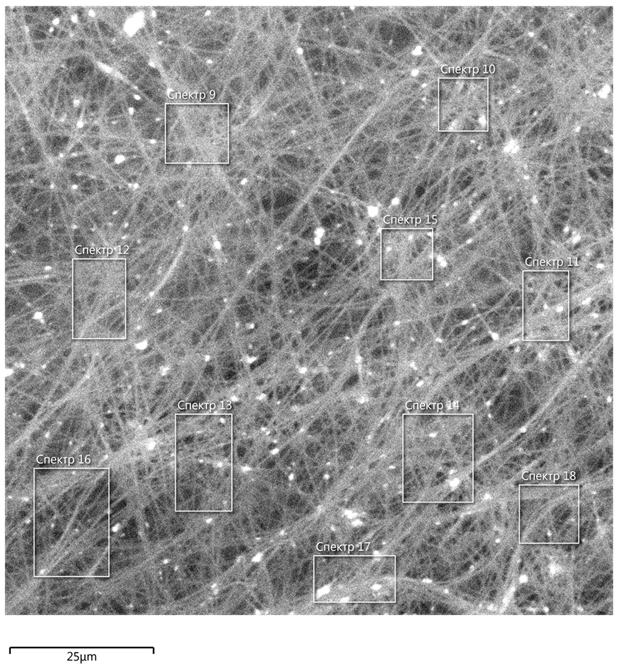

Supplement: Supplementary file 1 [file polymers-17-01255-s001.zip › Figure_S5.png]

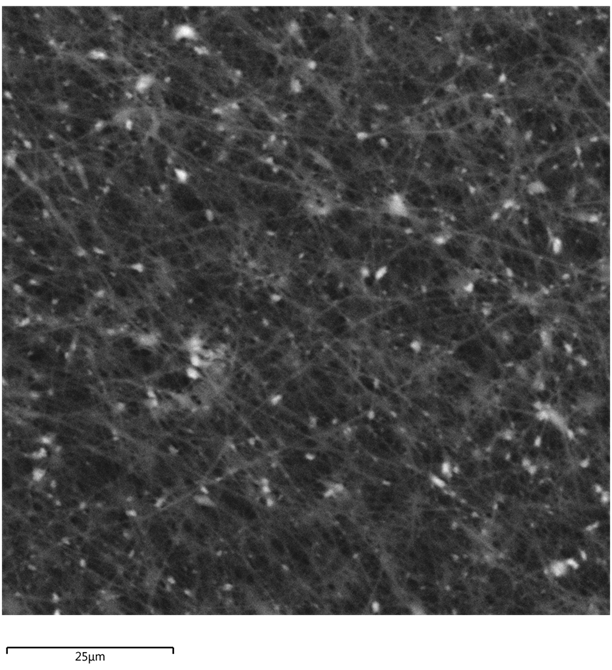

Supplement: Supplementary file 1 [file polymers-17-01255-s001.zip › Figure_S6.png]

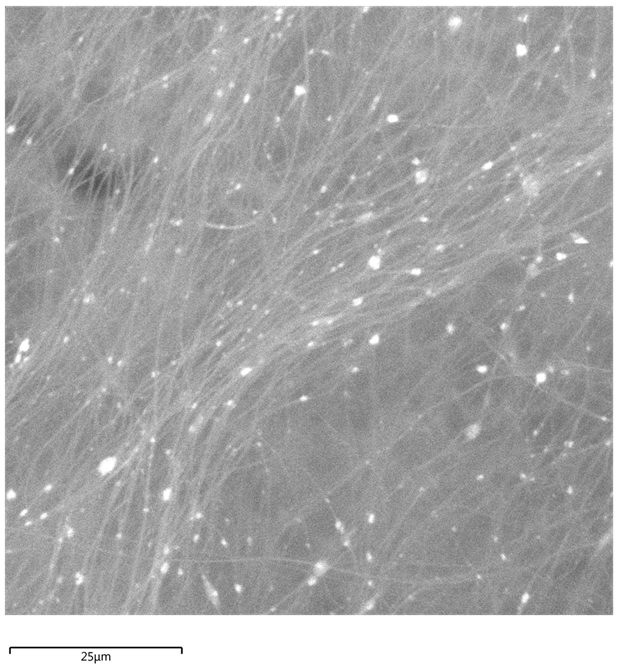

Supplement: Supplementary file 1 [file polymers-17-01255-s001.zip › Figure_S7.png]

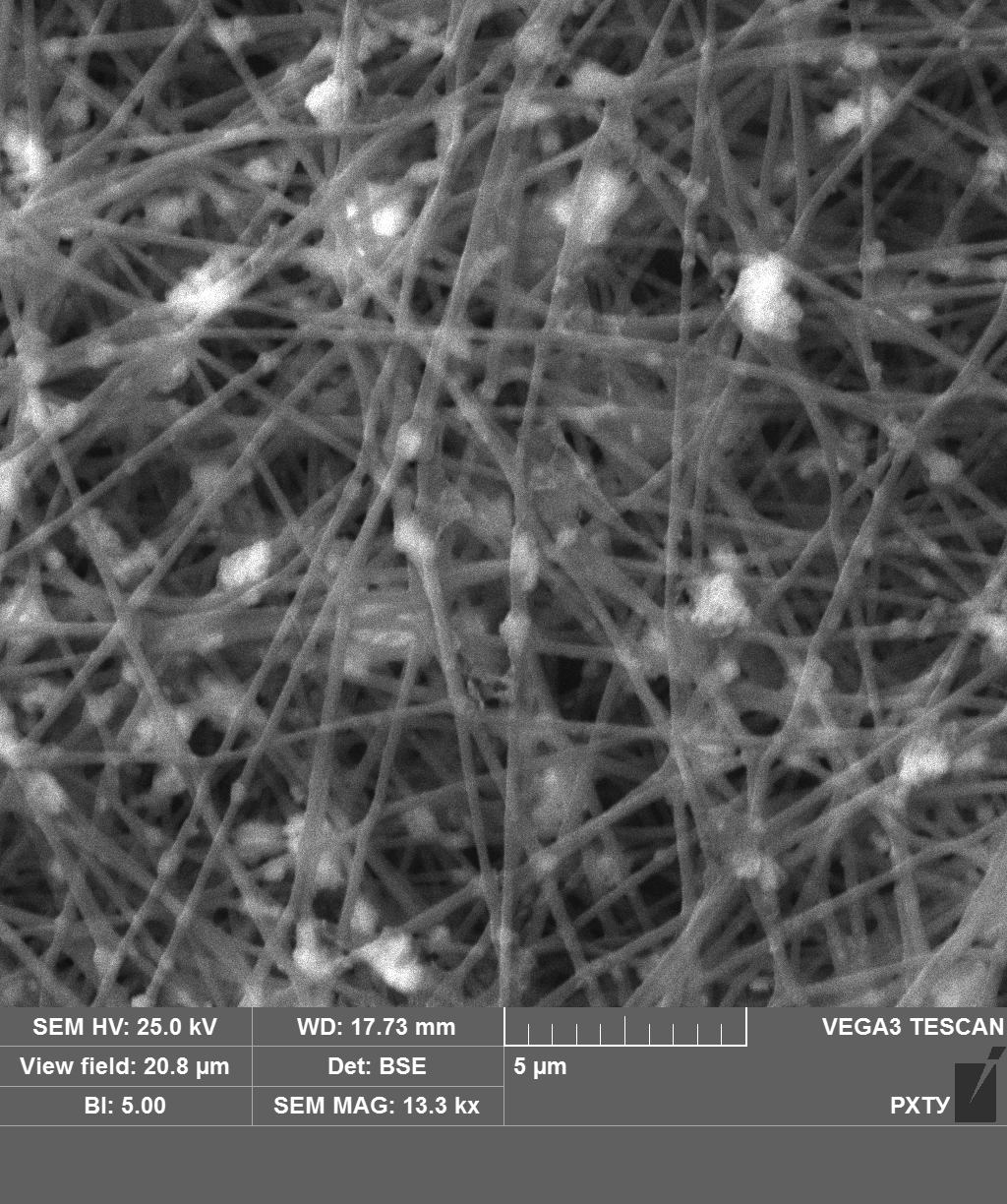

Supplement: Supplementary file 1 [file polymers-17-01255-s001.zip › Figure_S8.png]
